# Predictors of malnutrition among older residents in Qatari long-term care facilities: a retrospective study

**DOI:** 10.1186/s40795-024-00827-z

**Published:** 2024-02-01

**Authors:** Al Anoud Ali H. Z. AlFehaidi, Shafi Hashmath Ulla Khan, Rana Albdeljubbar Abdelrahman, Nesreen Talal Ahel, Pavithra Shine, Monica Doroja De Ramos, Nisreen Mazin Skairjeh, Shakeel Ahmad Khan, Reem Khalid Al-Saadi

**Affiliations:** 1 Department of Dietetics & Nutrition, Rumailah Hospital, Hamad Medical Corporation, Al Khaleej Street, Doha, P.O. Box: 3050, Qatar; 2 Department of Geriatrics & Long-Term Care, Rumailah Hospital, Hamad Medical Corporation, Doha, Qatar; 3 Department of Dietetics & Nutrition, Hamad Medical Corporation, Doha, Qatar

**Keywords:** Malnutrition, Older adults, Long-term care facilities (LTCFs), Predictors, Qatar

## Abstract

**Background:**

Malnutrition is a prevalent issue among older adults in long-term care facilities and is associated with adverse health outcomes and increased healthcare costs. Identifying the predictors of malnutrition in this population is crucial for developing effective intervention strategies. This study aimed to explore the factors contributing to malnourishment among older individuals living in long-term care facilities in Qatar.

**Methods:**

This cross-sectional study included 75 older adults from two long-term care facilities (Rumailah Hospital and Enaya Specialized Care Center) in Qatar. Baseline characteristics, including age, sex, length of stay, mortality, weight, body mass index, co-morbidities, and laboratory parameters, were assessed. Data were analyzed using the most recent version of the SPSS software, version 29. Predictors of malnutrition and mortality were identified using logistic regression analysis.

**Results:**

Of the 75 older individuals included in the study, 85% (64) were malnourished. The average age of the participants was 74.89 years, with a standard deviation of 10.21. Of all participants, approximately 61% (46) were males, and 39% (29) were females. Most malnourished older adults were classified as either at “moderate (29.69%)” or “severe risk (37.50%),” according to the Geriatric Nutritional Risk Index. Malnourished participants experienced a significant percentage of weight change within 3 months (14.01 ± 7.89); the only statistically significant predictor of malnutrition was the percentage of weight change within 3 months with an odds ratio (OR) of 4.8 (confidence interval [CI] 1.56–14.75) and *p*-value of 0.006. Statistically significant predictors of mortality were malnutrition (OR 24.84, CI 1.09–564) and age (OR 1.07, CI 1.00–1.14).

**Conclusions:**

A significant predictor of malnutrition in older adults identified in this study was the sudden and recent change in weight, which can be employed to detect individuals at risk early and guide tailored interventions. Malnutrition is a significant predictor of mortality. Employing a multidimensional strategy to tackle malnutrition can improve outcomes for the older individuals.

## Background

Malnutrition is a prevalent and detrimental health issue that affects millions of older adults worldwide, particularly those living in long-term care facilities (LTCFs). It is prevalent in this setting, with a prevalence rate of up to 60% [1–5]. The consequences of malnutrition in this population are significant and include increased morbidity [4], impaired functional status [6], extended hospital stays [7], increased cost burden [3], and high mortality rates [4, 8].

Numerous factors have been identified as potential predictors of malnutrition in older adults. These include aging [9, 10], which can lead to decreased appetite [11], changes in taste and smell, and difficulty in chewing and swallowing [12]; male sex—due to inability to fulfill higher energy requirements and behavioral factors; co-morbidities such as coronary heart disease, dementia, and chronic kidney disease (CKD) [9]; functional status impairment [13–15], such as difficulty in walking, dressing, and bathing; cognitive impairment, possibly hindering older adults’ ability to eat and drink independently; physiological parameters, such as weight changes and albumin levels [16]; social isolation and self-neglect [17], which may lead to decreased appetite and poor food intake; and economic factors—older adults may not be able to afford nutritious food owing to monetary constraints or unemployment [18].

In Qatar, a previous study found that the prevalence of malnutrition among older residents in LTCFs was 85% [1]. Across the Middle East/North Africa region, studies have shown high malnutrition rates ranging from 25 to 60% in various older populations and care settings [4, 16, 20, 21]. The high prevalence in Qatar and the region may be attributed to the participants’ complex co-morbidities and disabilities, despite receiving regular input from a multidisciplinary team [1]. Identifying the particular factors that could forecast outcomes is crucial to customize interventions that can effectively tackle the distinct difficulties faced by older individuals residing in LTCFs and enhance their nutritional status. This study aimed to identify the risk factors that lead to malnutrition, establish its prevalence, and assess how such risk factors were related to malnutrition among older individuals residing in LTCFs in Qatar.

## Methods

### Study design

This cross-sectional study was conducted at two LTCFs in Qatar - Rumailah Hospital and Enaya Specialized Care Center, which had a total bed capacity of 200. Older people residing in LTCFs have multiple co-morbidities, including diabetes mellitus, hypertension, stroke, anoxic brain damage, and cardiovascular diseases, with the majority of them bedbound and on artificial feeding.

Inclusion criteria included adults aged ≥ 60 years who were admitted to LTCFs between January 1, 2016 and December 31, 2018. Patients who were undergoing hemodialysis for end-stage renal disease; presented co-morbidities such as cirrhotic liver disease, autoimmune disorders, chronic neuromuscular conditions, or active cancer; or were under palliative care were excluded from the study to ensure a more homogeneous participant group, reduce confounding variables, and enhance the study’s internal validity. LTCFs provide care to residents with an age range of 18–104 years, with diverse and complex medical conditions like ESRD on HD and palliative care needs. Considering the previous study’s wide malnutrition prevalence range (25–85%) and complex patient profiles, meeting the sample size calculation was unfeasible. Therefore, all eligible patients meeting the inclusion criteria from January 2016 to the end of 2018 (3 years) were included, representing 37.5% of the LTCF’s population. The study encompasses 75 out of 200 residents.

### Data collection

Data was obtained by exploring electronic medical records using a comprehensive data collection sheet. Information regarding the participants’ demographics, medical background (medical and surgical history), skin integrity, nutritional status, functional status, oral health, fall risk, gastrointestinal symptoms, dysphagia evaluation, social history, and relevant laboratory parameters (such as the serum levels of bilirubin, glycated hemoglobin [HbA1c], calcium, and C-reactive protein [CRP]) was collected.

Nutrition assessment data electronically recorded during admission, including anthropometrics, body composition analysis (measured by body mass index [BMI], Geriatric Nutritional Risk Index [GNRI], and Global Leadership Initiative on Malnutrition [GLIM]), dietary intake, nutrition requirements, and nutrition care process were also collected. Complications of malnutrition and clinical outcomes, such as pressure injury occurrence, infections (urinary tract infection [UTI] and pneumonia), hip fracture rates, length of stay in LTCFs, and mortality, were recorded.

### Malnutrition definition

The European Society of Clinical Nutrition and Metabolism (ESPEN) [19] definition was used to define malnutrition. Malnutrition confirmation criteria included a BMI below 18.5, or a combination of unintentional weight loss over 10% of body weight at any time or exceeding 5% within the past 3 months, and a BMI < 20 for individuals < 70 years and < 22 for those ≥ 70 years. BMI was determined using weight and height (kg/m^2^). Weight loss percentage was calculated by comparing admission weight with weights from 3 to 6 months prior, sourced from medical records. These measures indicated whether participants met the ESPEN criteria for malnutrition. Participants were classified as malnourished if any BMI or weight loss criteria were met. This malnutrition definition was uniformly applied to all study participants.

### Statistical analysis

The statistical software SPSS version 29.0 (IBM, Chicago, USA) was used to analyze the data. Descriptive statistics were used to summarize the collected data. To evaluate the normality of distribution for continuous variables, the Kolmogorov–Smirnov test was used. Additionally, bivariate analysis was conducted to gauge any potential association between risk factors and malnutrition, whereas multivariate logistic regression analyses were used to identify independent risk factors for both malnutrition and mortality. Statistical significance was ascertained using a two-tailed *p*-value. A post-hoc power analysis was conducted using a general linear model with repeated measures. The observed power for malnutrition and mortality, both dependent variables, was determined to be 1, with a significance level of less than 0.001.

## Results

Seventy-five older adult participants were included in the study, 85% (64 individuals) of whom were malnourished.

Table 1 shows the baseline characteristics of the study population. The average age of the participants with malnutrition was 74.48 years (± 9.54), compared to the 77.27 years (± 13.81) of those who were non-malnourished, without a significant difference in age between the two groups (*p* = 0.40). In terms of sex, no notable variation was identified between the malnourished and non-malnourished groups (60.94% and 63.64% males, respectively; *p* = 0.86). Additionally, regarding co-morbidities, individuals with malnutrition had a lower prevalence of CKD than did those without (6.25% vs. 27.27%, *p* = 0.02). However, no significant differences in the presence of dementia (*p* = 0.24) or diabetes mellitus (*p* = 0.63) were found between the two groups.

**Table 1:** Baseline characteristics (*n* = 75)

Characteristics	Malnourished participants (*n* = 64)			Non-malnourished (*n* = 11)			*P* value
	Number	Percentage (%)	Confidence Interval	Number	Percentage (%)	Confidence Interval	
Age in years mean ± SD	74.48 ± 9.54	-	72.10–76.87	77.27 ± 13.81	-	67.99–86.55	0.40
Males	39	60.94	47.93–72.90	7	63.64	30.79–89.07	0.86
Females	25	39.06	27.10–52.07	4	36.36	10.93–69.21	
CKD	4	6.25	1.73–15.24	3	27.27	6.02–60.97	0.02
Dementia	23	35.94	24.32–48.90	6	54.55	23.38–83.25	0.24
DM	34	53.13	40.23–65.72	5	45.45	16.75–76.62	0.63
BMI < 23	22	34.38	22.95–47.30	5	45.45	16.75–76.62	0.66
BMI 23–25	26	40.63	28.51–53.63	5	45.45	16.75–76.62	
BMI 25–29	4	6.25	1.73–15.24	0	0	0	
BMI ≥ 30	12	18.75	10.06–30.46	1	9.09	0.23–41.28	
GNRI– No risk	8	12.5	5.55–23.15	0	0	0	0.06
GNRI– Low	13	20.31	11.28–32.23	6	54.55	23.38–83.25	
GNRI– Mod risk	19	29.69	18.91–42.42	1	9.09	0.23–41.28	
GNRI– Severe risk	24	37.50	25.70–50.49	4	36.36	10.93–69.21	
Percentage of weight change over 3 months (%) mean ± SD	14.01 ± 7.89	-	12.04–15.98	2.48 ± 1.01	-	1.79–3.16	< 0.01
Bilirubin (umol/L) mean ± SD	8.36 ± 4.68	-	7.19–9.5	14.28 ± 18.74		1.68–26.87	0.03
HbA1c (%) mean ± SD	7.76 ± 2.06	-	7.25–8.28	6.58 ± 1.58	-	5.51–7.64	0.07
Calcium (mmol/l) mean ± SD	2.39 ± 0.19	-	2.35–2.44	2.51 ± 0.10	-	2.44–2.57	0.07
CRP (mg/l) mean ± SD	66.08 ± 68.23	-	49.04–83.13	32.92 ± 26.56	-	15.07–50.77	0.11
Pressure injury	14	21.88	12.51–33.97	11	14.67	7.56–24.73	0.78
Stage 1 pressure injury	0	0	0	0	0	0	0.40
Stage 2 pressure injury	4	28.57	8.39–58.10	1	50	1.26–98.74	
Stage 3 pressure injury	3	21.43	4.66–50.80	1	50	1.26–98.74	
Stage 4 pressure injury	7	50	23.04–76.96	0	0	0	
UTI	23	35.94	24.32–48.90	2	18.18	2.28–51.78	0.24
Pneumonia	24	37.50	25.70–50.49	6	54.55	25.70–50.49	0.28
Hip fracture	15	23.4	13.75–35.69	7	63.64	30.79–89.07	0.07
Length of stay (Days) mean ± SD	203 ± 255	-	139–267	530 ± 472	-	212–848	0.001
Mortality	12	18.75	10.08–30.46	2	18.18	2.28–51.78	0.96

**Abbreviations**: BMI, body mass index; CKD, chronic kidney disease; CRP, C-reactive protein; DM, diabetes mellitus; GNRI, Geriatric Nutritional Risk Index; HbA1c, glycated hemoglobin; UTI, urinary tract infection

The BMI category division showed that 34.38% of malnourished individuals had a BMI < 23, compared to 45.45% in the non-malnourished group (*p* = 0.66). Moreover, 40.63% of the malnourished group had a BMI ranging from 23 to 25, whereas the parallel percentage for their non-malnourished counterparts was 45.45% (*p* = 0.66). A considerable number of the participants (*n* = 43, 67%) who had malnutrition were labeled either as having a “moderate risk (n = 19, 29.69%)” or “severe risk (n = 24, 37.50)” according to the GNRI; conversely, only five individuals from the group without malnutrition were identified as having a “moderate-severe risk” by the same index. The differences in the GNRI classification between the two groups were trending toward statistical significance (*p* = 0.06). Further analysis revealed that 20.31% of the participants with malnutrition were classified as presenting “low risk” by the GNRI, while 54.55% of the non-malnourished group fell into this category.

Biomarkers showed that older individuals who were malnourished had lower bilirubin levels (mean, 8.36 ± 4.68) than did those who were non-malnourished (mean, 14.28 ± 18.74); a statistically significant variation was observed (*p* = 0.03). No significant differences were identified in HbA1c (malnourished: 7.76 ± 2.06; non-malnourished: 6.58 ± 1.58, *p* = 0.07), calcium (malnourished: 2.39 ± 0.19; non-malnourished: 2.51 ± 0.10, *p* = 0.07), or CRP levels (*p* = 0.11).

The occurrence of pressure injuries did not significantly vary between the malnourished (21.88%) and non-malnourished groups (14.67%, *p* = 0.78). Older individuals who were malnourished had a higher prevalence of stage 4 pressure injuries (50%) than did non-malnourished ones (0%); the observed variation was not statistically significant (*p* = 0.40). No significant difference was found in relation to infections, namely UTIs (*p* = 0.24) and pneumonia (*p* = 0.28). Notably, the malnourished group had a lower prevalence of hip fractures (23.4%) than did the non-malnourished group (63.64%, *p* = 0.07). The length of stay was significantly shorter in the malnourished group (203 ± 255 days) than in the non-malnourished group (530 ± 472 days, *p* = 0.001). An equivalent observation was made in both the malnourished and non-malnourished groups, with mortality rates of 18.75% and 18.18%, respectively, without statistically significant differences between the two groups (*p* = 0.96).

**Fig. 1:**
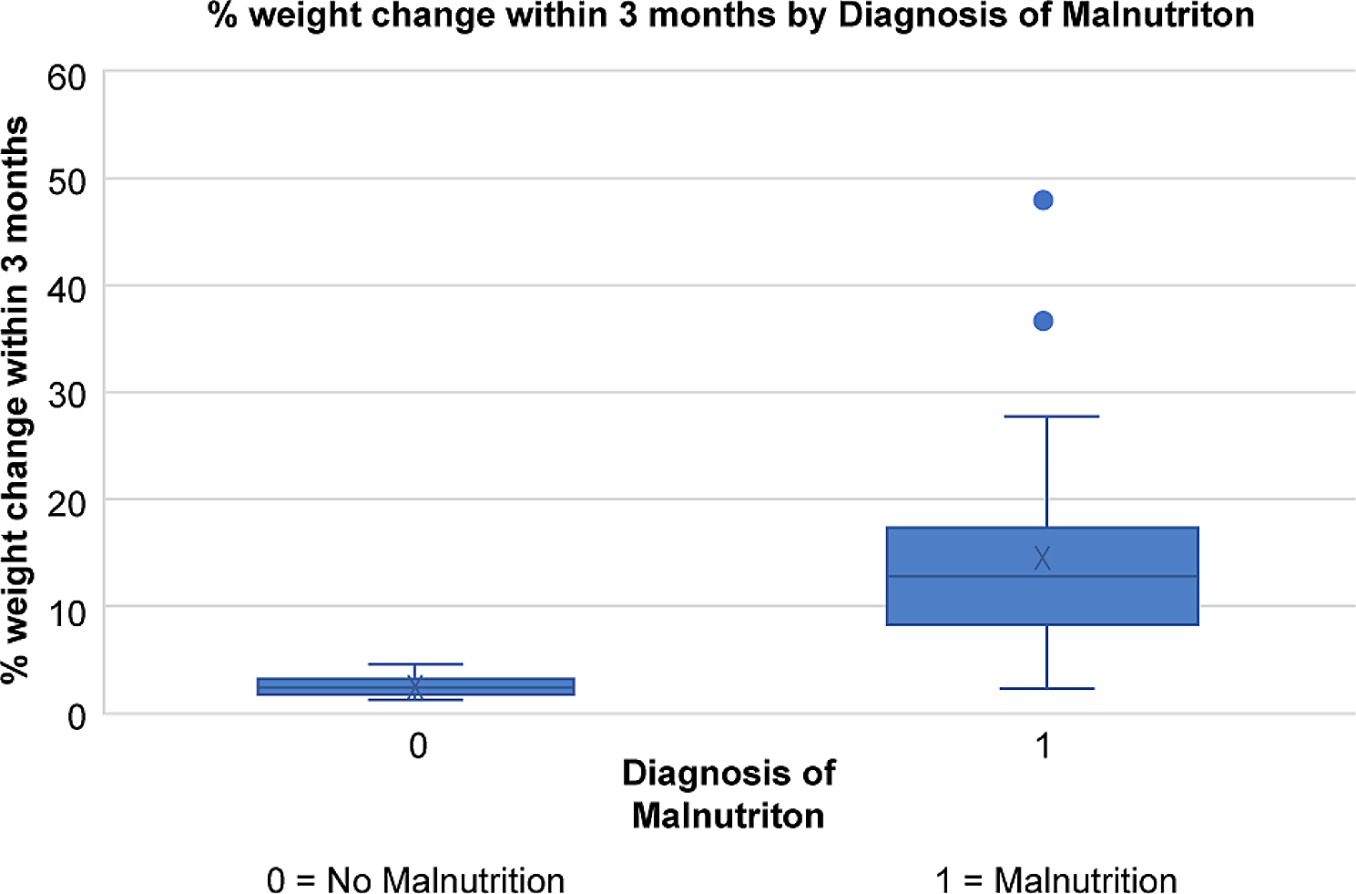
Percentage weight change within 3 months between the malnourished and non-malnourished groups. The percentage weight change between the two groups is presented as simple box plots. The “No Malnutrition” boxplot shows weight changes clustered tightly around the median of 2.1818, with an IQR of 1.25–3.03 and whiskers of 1.2–4.386. In contrast, the “Malnutrition” boxplot has a lower IQR of 8.17–16.81 and whiskers of 2.3327.43, indicating more variability. Outliers were observed at 36.42 and 47.83. The long upper tail shows that some participants with malnutrition experienced significant weight loss. IQR, interquartile range

A box plot diagram (Fig. 1) shows the distribution of weight changes between the two groups (No Malnutrition vs. Malnutrition). The “No Malnutrition” box has its lower whisker at 1.2, lower edge at 1.7544, median line at 2.1818, upper edge at 3.0303, and upper whisker at 4.386, indicating that the weight changes for the participants who did not have malnutrition are more tightly clustered around the mean. In contrast, the “Malnutrition” box has its lower whisker at 2.3256, lower edge at 8.1667, median line at 12.8815, upper edge at 16.8079, and upper whisker at 27.4285. Outliers were observed at 36.4161 and 47.8261. The box plot for participants with malnutrition had a long tail, indicating that some participants had experienced significant weight loss.

Table 2 outlines the outcomes obtained from the multivariate logistic regression analysis to identify potential predictors of malnutrition. Only the percentage of weight change within 3 months was statistically significant (*p* = 0.006). The odds ratio (OR) was 4.8 with a CI of 1.56–14.75, indicating that for every 1% increase in the percentage of weight change within 3 months, the odds of malnutrition increased by 4.80 times. Therefore, recent rapid weight loss appears to be a major risk factor for malnutrition in this population. This aligns with the significant difference in mean 3-month weight change seen between non-malnourished and malnourished groups. Diabetes was not a statistically significant predictor of malnutrition (OR: 4.57, *p* = 0.37, CI: 0.16–130), despite some research suggesting an association.

**Table 2:** Logistic regression (multivariate) analysis– malnutrition

Characteristics	Beta (unstandardized)	Standard error	Odds ratio	Probability value	95% confidence interval for odds ratio
Percentage of weight change within 3 months	1.57	0.27	4.80	0.006	1.56–14.75
DM	1.52	1.71	4.57	0.37	0.16–130.77

**Abbreviation**: DM, diabetes mellitus

The findings of the logistic regression analysis aimed at identifying the risk factors associated with mortality (Table 3). Malnutrition was found to be a highly significant predictor of mortality, with malnourished participants having nearly 25 times higher odds of mortality (OR 24.84, *p* = 0.04, CI 1.09–564). This highlights the severe consequences of malnutrition on outcomes in this population. Age was also a significant predictor, with higher age associated with slightly increased odds of mortality (OR 1.07, *p* = 0.04, CI 1.00–1.14). This reflects the impact of advanced age on mortality risk. Length of stay predicted mortality as well, though the odds ratio was close to 1, indicating a minor effect (OR 1.00, *p* = 0.01, CI 1.001–1.006). Sex was not a significant predictor of mortality (*p* = 0.21). This contrasts with some research indicating sex differences in malnutrition prevalence and mortality. Recent weight loss percentages did not significantly predict mortality (*p* = 0.08), despite the significant differences seen between nourished and malnourished groups. This suggests other factors may mediate the relationship between weight loss and death.

**Table 3:** Logistic regression (multivariate) analysis– mortality

Characteristics	Beta (unstandardized)	Standard error	Odds ratio	Probability value	95% confidence interval for odds ratio
Age	0.71	0.35	1.07	0.04	1.00–1.14
Sex	1.02	0.82	2.77	0.21	0.55–13.97
Percentage of weight change within 3 months	-0.11	0.06	0.89	0.08	0.78–1.01
Malnutrition	3.21	1.59	24.84	0.04	1.09–564.29
Length of stay	0.003	0.00	1.00	0.01	1.001–1.006

## Discussion

In our study, the prevalence of malnutrition was exceedingly concerning, as 85% of the participants were categorized as malnourished; this aligns with previous research that has shown similarly high rates of malnutrition among older adult populations in the Middle East/North Africa region [16, 20, 21]. Additionally, the high prevalence in this study may be attributed to the participants’ complex co-morbidities and disabilities, despite receiving regular input from a multidisciplinary team [1].

Despite previous studies linking a few co-morbidities to malnutrition, our study did not identify any specific comorbid predictors of malnutrition [9]. Our study found that hip fractures trended toward statistical significance as predictors of malnutrition, but the association did not reach the threshold for significance (*p* = 0.07). Previous research has shown that hip fractures can negatively affect nutritional status, leading to greater dependence on others and higher mortality rates [22]. Hip fractures frequently cause reduced mobility and functional limitations, resulting in decreased appetite, increased energy needs, and impaired nutrient intake. Conducting early nutritional assessments and interventions, as well as rehabilitative measures, is critical to promote optimal nutrition and recovery in this population.

BMI has become an efficient tool to quickly and conveniently detect malnourishment and is widely regarded as a more favorable assessment technique than other approaches [23, 24]. However, some disadvantages are associated with BMI. For example, values may take longer to reflect reduced food intake and biochemical or inflammatory markers are not considered during the assessment [25]. The importance of using nutritional risk assessment tools in long-term care is well established. GNRI is a useful tool for identifying the risk of malnutrition and has been successfully applied in various settings, including long-term care. It is particularly effective in predicting functional status and mortality [26]. In our study, a variance in the GNRI risk was identified among malnourished and non-malnourished individuals. Although such differences indicated a tendency toward statistical significance, no correlation between the GNRI and mortality was found in our study.

The link between malnutrition and the percentage of weight change within 3 months was found to be strong in our study, identifying such as an important predictor. Similar observations have been made in previous studies [27, 28]. Rapid weight loss may indicate underlying health problems, insufficient intake of nutrients owing to loss of appetite [11], or alterations in metabolic processes. Regularly monitoring weight and implementing timely intervention measures, such as individualized nutritional plans and supplementation, is essential to prevent and manage malnutrition [29].

The identification of mortality indicators is an ongoing pursuit aimed at decreasing mortality rates. A strong association has been established between malnutrition and an increased mortality risk [28, 30]. Individuals who are malnourished have impaired immune function, wound healing, and muscle strength. As a result, the underlying diseases and complications can become more severe, which can make this population more vulnerable to infections, lengthen their recovery period, and increase their chances of treatment failure. Ultimately, this increases the mortality risk. Moreover, malnutrition often occurs alongside chronic conditions such as cancer, cardiovascular disease, and respiratory disorders. This worsens the negative consequences of these conditions and creates a cycle in which poor nutritional status leads to the further deterioration of health outcomes caused by the underlying disease. Our study indicated a strong association between malnutrition and mortality. Patients with malnutrition were approximately 25 times more susceptible to mortality, as indicated by an OR of 24.84; this significant increase in mortality risk highlights the crucial effect of malnutrition on patient outcomes. Of note, the confidence interval has a broad range owing to the sample size and possible variations within the patient group, indicating that our estimate may be imprecise. Nonetheless, our study implies that efficiently identifying and addressing malnutrition is crucial for minimizing the adverse consequences in this susceptible population.

Our study, with the discovery of a high prevalence of malnutrition despite rigorous nutritional assessment and management, emphasizes the necessity for a multidimensional approach in Qatar’s long-term care settings. Combining nutrition, physical therapy, medication management, swallowing therapy, and medical care is likely necessary to enhance outcomes. The percentage of weight change within 3 months was identified as the only statistically significant predictor of malnutrition risk in older Qatar LTCF residents. This underscores the significance of vigilant monitoring for body weight fluctuations, serving as an early warning system for potential malnutrition risk. LTCFs could adopt weekly or biweekly weight assessments and establish thresholds to initiate nutrition consultations and interventions in the event of abrupt weight loss. Offering high-protein and calorie-dense foods, coupled with daily activity assistance, can address heightened nutritional requirements during rehabilitation. Moreover, any major health changes, such as acute illnesses, should prompt an evaluation of nutritional status. If malnutrition is identified, individualized nutrition intervention plans should be immediately implemented, focusing on altering modifiable risk factors. This may involve diet liberalization to allow favorite foods, nutrient-dense meal and snack options, oral nutrition supplements, and feeding assistance as needed. Although no specific co-morbidities were predictive in this study, prior research suggests that conditions such as dementia and chronic kidney disease elevate the risk of malnutrition. Therefore, conducting screenings to assess the overall co-morbidity burden and associated nutritional risks is advisable.

### Study limitations

This study has several limitations. First, it had a cross-sectional design, which prevented the establishment of causality between the variables. Second, we used self-reported data, which may not be entirely accurate and could have introduced bias. Additionally, the sample size was small, which limited the generalizability of the findings to a larger population. There is also the possibility of selection bias, as the sample may not represent the target population. Finally, all the limitations and potential biases associated with this research design apply to this study as well.

## Conclusions

Targeted interventions are urgently needed to address malnutrition owing to its high prevalence. Although there are many established predictors for the condition, our study reiterates the percentage of weight change within 3 months as one of the most important. These findings demonstrate the critical significance of treating malnutrition as part of patient care, emphasizing the necessity of comprehensive nutritional assessments and tailored interventions to optimize results and decrease mortality rates in vulnerable populations.

## Data Availability

The data that support the findings of this study are from the Medical Research Center of Hamad Medical Corporation. However, restrictions apply to the availability of these data, which were used under license for the current study and are not publicly available. Nevertheless, data are available from the authors upon reasonable request and with permission from Hamad Medical Corporation.

## Abbreviations

LTCFs: Long-term care facilities
BMI: Body mass index
GNRI: Geriatric Nutritional Risk Index
HbA1c: Glycated hemoglobin
CRP: C-reactive protein
UTI: Urinary tract infection
ESPEN: European Society of Clinical Nutrition and Metabolism
CKD: Chronic kidney disease
OR: Odds ratio
